# Evaluation of partnerships in a transnational family violence prevention network using an integrated knowledge translation and exchange model: a mixed methods study

**DOI:** 10.1186/1478-4505-12-25

**Published:** 2014-05-23

**Authors:** Anita Kothari, Shannon L Sibbald, C Nadine Wathen

**Affiliations:** 1 Faculty of Health Sciences, Western University, N6A 5B9 London, ON, Canada; 2 Faculty of Information and Media Studies, Western University, N6A 5B7 London, ON, Canada

**Keywords:** Family violence, Integrated knowledge translation, Networks, Partnerships, Public health

## Abstract

**Background:**

Family violence is a significant and complex public health problem that demands collaboration between researchers, practitioners, and policymakers for systemic, sustainable solutions. An integrated knowledge translation network was developed to support joint research production and application in the area. The purpose of this study was to determine the extent to which the international Preventing Violence Across the Lifespan (PreVAiL) Research Network built effective partnerships among its members, with a focus on the knowledge user partner perspective.

**Methods:**

This mixed-methods study employed a combination of questionnaire and semi-structured interviews to understand partnerships two years after PreVAiL’s inception. The questionnaire examined communication, collaborative research, dissemination of research, research findings, negotiation, partnership enhancement, information needs, rapport, and commitment. The interviews elicited feedback about partners’ experiences with being part of the network.

**Results:**

Five main findings were highlighted: i) knowledge user partner involvement varied across activities, ranging from 11% to 79% participation rates; ii) partners and researchers generally converged on their assessment of communication indicators; iii) partners valued the network at both an individual level and to fulfill their organizations’ mandates; iv) being part of PreVAiL allowed partners to readily contact researchers, and partners felt comfortable acting as an intermediary between PreVAiL and the rest of their own organization; v) application of research was just emerging; partners needed more actionable insights to determine ways to move forward given the research at that point in time.

**Conclusions:**

Our results demonstrate the importance of developing and nurturing strong partnerships for integrated knowledge translation. Our findings are applicable to other network-oriented partnerships where a diversity of stakeholders work to address complex, multi-faceted public health problems.

## Background

To bridge so-called ‘knowledge-practice gaps’ in health care policy and practice, the field of knowledge translation (KT) has evolved tremendously from its inception approximately a decade ago. While earlier conceptualizations of KT focused on mechanisms to effectively transfer knowledge from researchers to knowledge users, the scope of who constitutes ‘knowledge users’, initially constructed as health care providers (mainly physicians), now encompasses a range of knowledge users, from policymakers to patients and members of the public. In addition, more recent theories have evolved to recognize that barriers in the ‘knowledge to action’ process may lie not only in knowledge user capacity and research dissemination mechanisms, but in the knowledge production process itself [1]. It has been acknowledged that there may be a disconnect between two very different communities that have different needs, priorities, and conceptualizations of knowledge [2-4]. KT strategies that adhere to this more nuanced approach, including integrated knowledge translation (IKT) [1,5], focus on two-way interactions between researchers and knowledge users [6], i.e., ‘exchange’.

IKT engages both researchers and knowledge-users in the entire research process, from definition of research questions to dissemination of research results [7]. It is meant to be dynamic, collaborative, and to cross disciplinary boundaries [8]. An essential element of a well-implemented IKT process is a successful partnership [9] between all groups involved, which can represent diverse sets of actors, including researchers, community groups, policymakers, advocates, and health and social service providers. Partnerships in public health are becoming increasingly normative, through networks, coalitions, and formalized agreements between different institutions (e.g., the Pan-Canadian Public Health Network [10]), including international networks to address such issues as infectious disease transmission [11]. However, partnerships for the explicit purpose of IKT have not been deeply explored in the academic literature [11,12].

Conceptual work regarding the functioning of IKT-oriented public health partnerships is scant. Clavier et al. proposed that knowledge brokers or intermediary agents/organizations use three practices to facilitate KT in public health partnerships: cognitive, strategic, and logistic [13]. Cognitive practices ensure that knowledge of all partners is known and used, and contributes to activities such as shared formation of research questions and knowledge production. The purpose of strategic practices is to maintain the interest of partners in the research process, and can involve activities or tools for partner engagement. Logistic practices are the day-to-day activities that actually bring the partnership together, such as coordinating meetings and other joint activities. While this construct contributes to understanding of IKT-oriented partnerships, it is specific to knowledge brokers – a role that many partnership structures do not include.

More is known about partnership characteristics and processes that are needed for high-quality and ultimately successful relationships. Trust and respect between all parties, built over time, are essential [11,14-19] for an equitable, collaborative partnership [9]; these require sustained contact, preferably including face-to-face meetings. All partners should also share a common language [20] and have common goals [17,18,21] in order to unify the partnership’s purpose and increase a sense of commitment among members. It is important that there are clear roles and expectations [14] for all parties that accommodate different needs and capacities, and that leadership is inclusive [22]. More specific to KT activities, knowledge exchange should be a two-way process – it should be understood and accepted in the group that both knowledge-users and researchers bring different types of knowledge to the table and can learn from each other [23]. Not privileging one source or type of knowledge over another can help to maintain a balance of power between knowledge producers and users [24]. In this vein, it has been suggested that many different types of information should be included in the research process, for example local data or knowledge user experience as well as academic literature [25]. Products of the research partnership should be presented to all partners and disseminated in a way that is relevant, timely, and accessible to users [15,26].

Despite a wealth of information on what is needed for a successful partnership, there is a paucity of empirical research on how partnership quality and functioning translate into outcomes, and the research that does exist is inconclusive. A recent systematic review of 15 studies on public health partnerships that aimed to improve health in the UK found that there was no clear evidence on how these partnerships impacted health outcomes, and the study designs made it difficult to attribute successes and failures to partnership structures and processes [27]. A recently published Cochrane Review [28] examined 11 studies (randomized controlled trials, controlled before-and-after studies, and interrupted time series) to determine the effects of interagency collaboration between local health and local government agencies on health outcomes. Finding no evidence of health gain, one explanation put forth by the authors was “*that the process of collaboration may not have been optimal, leading to interventions not being fully delivered*” [28, p. 33]. Others agree that the ‘black box’ of collaborative efforts is still not fully understood [29], which has been attributed by some to shortcomings in traditional research methods used to study partnerships [30]. The evaluation of partnerships for the purpose of KT is an area that has received even less attention [31]. The purpose of our study was to determine the extent to which an international public health network built effective partnerships among its members, with a focus on the knowledge user partner perspective.

### The PreVAiL research network (http://www.PreVAiLResearch.ca)

In 2009, the PreVAiL (Preventing Violence Across the Lifespan) Research Network [32] – a 5-year federally-funded Centre for Research Development in Gender, Mental Health, and Violence Across the Lifespan – was established with three main objectives: i) to increase understanding and knowledge about the links between mental health impairment, substance abuse, gender, and exposure to child maltreatment and intimate partner violence, both in Canada and internationally; ii) to develop, using a resilience lens, interventions to prevent or reduce child maltreatment, intimate partner violence, and subsequent mental health problems; and iii) to develop and promote an integrated research and KT agenda among a network of established, new, and emerging investigators and key stakeholders. PreVAiL situates family violence as a major public health problem with impacts on health and well-being across the lifespan; its partners include agencies such as the World Health Organization, the US Centers for Disease Control and Prevention, and the Public Health Agency of Canada. It is therefore framed explicitly as a public health network, but with an interdisciplinary composition including researchers and partners from diverse areas within and outside of health (e.g., justice, child welfare, information science, etc.). The public health approach to violence attempts to understand a problem from its epidemiology, through to risk and protective factors, and intervention evaluation and implementation [33,34]. The Network is organized around two main content areas – child maltreatment and intimate partner violence against women – with a significant emphasis on mental health impacts of violence, and on how individuals develop resilience following exposure to these negative health outcomes.

PreVAiL is international in scope, with 60 researchers and knowledge-user partners, and 15 trainee members (Table 1) from Canada, the US, the UK, Asia, Europe, and Australia. Many of the partners represent organizations that either set policies, or advocate for and influence policy directions in a national and international context. Additional information about partner and researcher backgrounds can be found at http://www.PreVAiLResearch.ca. PreVAiL’s active approach involves partners in knowledge generation (where possible), dissemination, and the utilization of research findings associated with the program. The majority of PreVAiL’s funding is used to support attendance by all members (including partners) at team meetings, and to hold competitive rounds of project funding for ‘seed grants’ to launch research in PreVAiL’s priority areas, including KT-specific research. Activities are structured such that there are strong incentives or requirements (i.e., in the seed grants) to include partners (and trainees) in research and related activities, including priority-setting. That said, the Network is voluntary, and all members can opt in or out of specific activities. Strong partnerships are key for this research network to have an impact in practice. A successful IKT process ensures that relevant and timely research will be used by partners to develop effective policies, and in turn influence systems, around family violence and how it is addressed in our society.

**Table 1:** PreVAiL member breakdown

**Member type**	**Canadian (%)**	**International**^**1 **^**(%)**	**Total**
Researcher	26 (63.4%)	15 (36.6%)	41
Partner organizations^2^	12 (63.2%)	7 (36.8%)	19
New/emerging investigator^3^	13 (87%)	2 (13%)	15
**Total**	**51 (68%)**	**24 (32%)**	**75**

^1^The organization is international, or the individual works/studies at an international institution.

^2^Some partner organizations had more than one individual liaison.

^3^New/emerging investigators (graduate students, post-doctoral fellows, and new faculty) were added after Network initiation, and some ‘graduated’ as they completed their training programs; this was the number at time of data collection.

The purpose of our study was to determine the extent to which the PreVAiL network built, in its formative stages, effective partnerships among network members, with a focus on the knowledge user partner perspective.

## Methods

### Study design

The objectives of this mixed methods study were to examine the: i) quality and ii) initial impacts of the partnerships within the PreVAiL network. Data were collected in 2011 and 2012, two years after the network became operational. The University of Western Ontario’s Office of Research Ethics reviewed the project protocol and granted an ethics review waiver.

### Data collection

Data were collected through the Partnership Indicators Questionnaire (PIQ) [35] and semi-structured interviews. Indicators provide a transparent diagnostic checklist by which to guide the development of a partnership. The PIQ contains partnership indicators in the domains of communication, collaborative research, dissemination of research, research findings, negotiation, partnership enhancement, information needs, rapport, and commitment [35], and was adapted for this sample. Simple demographic questions were also included.

The questionnaire was administered at the PreVAiL annual team meeting in May 2011; time was allocated on the meeting agenda to provide details about the research and to allow those who wished to participate the time to fill out a paper copy of the questionnaire. Semi-structured interviews were conducted by phone from November 2011 to March 2012. The following areas were discussed to gain further insight into partners’ experiences with being part of PreVAiL, and their thoughts about what was going well and what could be improved: involvement with PreVAiL researchers and participation in formal PreVAiL activities or activities facilitated by the link with PreVAiL, as well as any active or passive sharing and/or use of knowledge arising from their relationship with PreVAiL.

### Sample

From the beginning the PreVAiL Network was conceived of as an IKT initiative, therefore reference to the ‘project team’ or ‘co-investigators’ encompasses trainees, partners, and researchers. In total, there were approximately 75 people on this project team, all of whom were invited to attend the annual meeting. Those present at the meeting (n = 57) were invited to complete the PIQ. All 22 partners (representing 19 partner organizations) were invited to participate in the semi-structured interviews, representing the knowledge user perspective.

### Analysis

As a first step, PIQ responses were analyzed in aggregate. Responses were then examined by ‘researcher’ (including researchers and trainees) and knowledge user ‘partner’ categories; data were also examined by type of knowledge user role categories (e.g., policymaking, advocacy). Frequencies were calculated for each indicator and domain.

All semi-structured interviews were audio-recorded and transcribed [36]. Transcript segments were coded inductively and care was taken to document operational definitions of each code. The trained data analyst and a research team member met after three transcripts were coded to ensure that code descriptions were clear and comprehensive. Then, after all transcripts were coded, another research team member reviewed all the coding to ensure trustworthiness of the process; discrepancies were resolved by discussion. Drawing on Braun’s approach [36], the entire research team met to interpret the coded data, leading to amalgamating, discarding, and identifying relationships among the codes (i.e., identify broader themes); over 200 initial codes were transformed into 14 themes with associated lower level codes. Descriptive data regarding specific activities and events were quantitatively summarized.

## Results

In total, 37/57 PreVAiL members at the May 2011 meeting completed the PIQ (65% response rate); one survey was omitted due to incomplete response (total sample n = 36). The majority of participants were researchers (including 9 trainees) (n = 26, 72% of sample). In total, 9 partners (25%) completed the PIQ. The primary activity of partners who participated was research/development (n = 6; 66%), followed by government policy development (n = 3; 33%), and advocacy (n = 1; 11%). One person (3%) did not provide demographic information.

In the following analysis ‘agree’ and ‘strongly agree’ are grouped together. We have also indicated in several places when the ‘not applicable’ selection was used by a large number of participants. Frequency tables are included in Additional file 1. With respect to the qualitative data, 19 interviews were conducted (86% response rate), representing 17 of our 19 partner organizations (Table 2).

**Table 2:** Summary of interviews

**Status**	**Number**
Invited	22 people from 19 PreVAiL partner organizations
Declined (role change)/Not completed in time	3
Total interviews	19 (of 22) (86%)
Total organizations represented	17 (of 19) (89%)

The results are presented in two sections. First, we present findings on the quality of the partnership, followed by a discussion of findings as they relate to the initial partnership impacts.

### Quality of the partnership

In order to understand the quality of partnerships, we focused on levels of partner involvement, quality of communication between network members, and value that partners found in their relationship to the network.

#### Levels of partner involvement

We asked partners about their participation in formal team-wide PreVAiL events and activities, as well as in formal and informal KT activities – a total of eight types, described and summarized in Table 3. The majority of partners participated in the face-to-face meetings in 2009 and 2011. Partners enjoyed these events and spoke highly of the benefits of networking, linkages, and meeting international researchers. Almost all partners said they would want more face-to-face meetings; while they recognized the expense incurred, the benefits to them were clear.

**Table 3:** Summary of activities/events by knowledge user partners

**Formal PreVAiL team activity**	**N (%)**
1. Nov 2009 Team Meeting	10 (53%)
2. May 2011 Team Meeting	14 (74%)
3. PreVAiL research priority-setting process (Delphi)	
3a. online survey 1	8 (42%)
3b. online survey 2	7 (37%)
*Completed at least one survey*	15 (79%)
*Completed both on* ***-*** *line surveys*	6 (32%)
3c. Child maltreatment teleconference	5 (26%)
3d. Intimate partner violence teleconference	2 (11%)
3f. Resilience teleconference	3 (16%)
*Participated in at least one teleconference*	9 (47%)
3g. Discussions at May 11 meeting	8 (42%)
*Participated in at least one component of the Delphi*	15 (79%)
**Formal and informal KT/linkage activities**	
4. Involved in a PreVAiL research project	2 (11%)
5. Formal or informal meetings with PreVAiL researchers (including having them speak to your organization; participate in panels, reviews, etc.)	12 (63%)
6. Interactions with PreVAiL trainees (outside of team meetings)	4 (21%)
7. Linking PreVAiL researchers with others in your organization	7 (37%)
8. Linking PreVAiL researchers with others in your broader professional network	9 (47%)
Other (specified by interviewee)	0

A PreVAiL Delphi research priority-setting process [37] took place from July 2010 to May 2011 and consisted of two survey rounds to identify and begin to rank priorities, a teleconference round (with three calls, one each for the theme areas child maltreatment, intimate partner violence, and resilience), and small and large group discussions at the May 2011 full-team meeting to finalize the ranked list of priorities by theme area. The on-line surveys had the highest participation rates, and nearly half of participants recalled participating in at least one teleconference; however, several were unsure of which specific call. Over half of the 14 partners who attended the May 2011 meeting recalled participating in the Delphi small group discussions.

When asked about other KT activities, almost two-thirds of partners talked about either formal or informal meetings with PreVAiL researchers (outside of team meetings). Only two partners mentioned they were involved with specific PreVAiL research projects; many were unaware of current projects. Similarly, there was a lack of activity around connecting with trainees; however, four participants did give successful examples of such linkages. Partners were more likely to talk about linking PreVAiL researchers with others in their organization, or in their broader professional network (Table 3).

In the interviews, many participants expressed their desire to be more involved with PreVAiL network activities such as collaborating on grants, research proposals, and joint advocacy on shared issues. A few participants simply wanted to become more aware of opportunities for involvement and broader networking on both a national and international level.

#### Quality of communication

All project team members were asked about their experience with PreVAiL’s communication processes (Table 4). On the PIQ, the majority of partners and researchers agreed that team communication is ongoing. Most participants used the same contact people for PreVAiL-related communications; however, this was truer for partners. This was echoed in the interviews where partners talked about contacting only one or two PreVAiL researchers with whom they were more familiar (often the same researcher who originally engaged them with PreVAiL) as a key point of contact.

**Table 4:** Communication dimension on the partnership indicators questionnaire (PIQ)

**Communication dimension**	**N (%) partners**	**N (%) researchers**
Communication is on-going	7 (78%)	16 (62%)
The same contact people continue over the life of the project	9 (100%)	14 (54%)
A common language/lexicon is used by all parties	2 (22%)	12 (46%)
Roles, expectations, and criteria for deliverables are explicit	4 (44%)	12 (46%)
Communication is frequent	4 (44%)	13 (50%)

Some other areas that showed discrepancies between researchers and partners were in the use of a common language and clarity around specific roles and expectations. While two partners agreed that a common language was used, three partners were undecided and another three disagreed. Researchers, on the other hand, were more in agreement that there was a common language. The majority of participants agreed that network members’ needs and constraints are expressed, but partners’ reactions were mixed: three agreed, and four were undecided (none disagreed). When asked about joint ongoing evaluation of relevance of research (e.g., current projects, new findings, new partner needs, etc.) the majority of participants agreed that evaluation is occurring, however, nearly an equal group said the question was not applicable.

Although the majority of participants agreed that communication was frequent, 13 of these respondents were researchers. Partners were more split: four felt that communication was frequent, and three were undecided. Six researchers and two partners felt communication was not frequent. Part of this variation in response could be due to a difference in understanding ‘frequent’. In the interviews, partners disagreed about what ideal communication would look like (both modality and frequency), however, most partners want to be more informed about PreVAiL activities. Some wanted more frequent communication (one partner suggested weekly emails), while others felt bi-monthly would be appropriate. A large number of partners wanted more information from PreVAiL on network activities including progress on seed funded projects, specific activities related to network objectives, and updated research findings.

Some partners wanted PreVAiL communication to also include more targeted or ‘tailored’ communication:

“*I actually expected minimal communication. And so* [what is currently being done] *meets my expectations, I’m not worried either way …, am I able to communicate with people when I need to, and I am, so that to me is the key thing because the main focus of PreVAiL is really child maltreatment and intimate partner violence, that’s not really my area; those are the main areas which generate communications, I’m not all that interested in receiving that information, I’m in information overload as it is. So when there’s something specific to the resilience construct, like I’m saying, I’m on that, that’s more what I’m interested in…*” [P-07]

One participant suggested that a communication plan would be helpful in both fostering better awareness of what is happening with the network, but also in allowing for more engagement with the network and its research.

“*…realizing that there’s some difficulties in making such a thing happen… it would be perhaps beneficial if there were an opportunity to understand from an expectation perspective what types of information you’re intending to communicate, the kind of timing of when it would be available. And the reason that I’m suggesting this is that if there’s something that is sort of important to respond to and you know, the information is being provided in a…passive way…that might be a way to sort of prime the pumps so to speak, so there’s some relationship to the process.*” [P-02]

#### Value of PreVAiL as a network

Another way to understand the quality of partnerships is to explore the value of the network for its partners. PreVAiL is perceived as an evolving research community whose people are essential to its success. Many of the partners value the ability to work with researchers committed to this content area. Networking, the key benefit reported by partners, has led to collaborations in writing papers, working on grants, and speaking at conferences/workshops. Even though primary research outcomes arising from PreVAiL studies were still forthcoming, the majority of respondents agreed that being a part of PreVAiL was helpful to them.

“[PreVAiL] *put me in contact with a lot of new people working in the area of violence prevention and these people have helped us with specific projects, these people actually carried out some work for us, they helped us get in touch with other people in other parts of the world, so people are networking and sources of help and people who can do work for us.*” [P-01]

Overall, team members seemed to value each other’s contribution within the network. In the PIQ, 75% said contributions were valued and 56% felt they were acknowledged in project documentation. This was especially true for partners: 78% felt both they valued each other’s contribution and felt they were acknowledged in project documentation. Most researchers agreed they were acknowledged in project documentation (50%), however, some (38%) saw this question as inapplicable.

When partner organizations’ goals were closely aligned with those of PreVAiL, participants spoke of the added value, ease of buy-in, and excitement for future collaboration. When asked why they accepted the invitation to join PreVAiL, one partner said: “*it fit so clearly with our mandate*” [P-05]. While not all partner organizations had the same goals as PreVAiL, nearly all reported on the importance of PreVAiL and the value of being part of the network. In this way, partners found value in getting together and working collectively:

“*What I valued was the chance to work with a group of committed people who were all very, seemed to be committed to these issues and that in itself was very, very valuable.*” [P-16]

When partner priorities or scope-of-work did not exactly match those of PreVAiL, it was not seen to be a problem or a weakness; participants were very ‘matter-of-fact’ about it: “*that’s just how it is.*” [P-01] and:

“*I’m not sure that the things we’re interested in are necessarily things that are on the top of your list, so I wouldn’t expect that there would be a great deal of information coming out from PreVAiL on certain topics that you’re not dealing with specifically.*” [P-10]

One participant went on to say that her organization is involved with many more topics than those that PreVAiL addresses, and she uses the connections from PreVAiL and the website as a resource in trying to sort out areas her organization might focus on. Participants also felt a key value of the network is that it is addressing important research areas that are often not a key focus on research agendas; this was a reason individuals wanted to be part of the network. In addition, partners had the opportunity to learn about new research and meet researchers, in face-to-face meetings, whose work was unfamiliar. In this way, partners were learning about new content and they found value in this. One participant stated that “*meetings are a catalyst for conversation*” [P-17], and the majority of participants called for more face-to-face meetings, potentially on a smaller scale.

### Impacts of partnerships within the PreVAiL network

This section explores how the PreVAiL network had an impact on its members in terms of the application of knowledge to policy and practice.

Partners used the PreVAiL network as a source for synthesized information. Some partners said they checked the website to get information; this was done most often when a partner had a specific need and knew related information was available on the website. In order to capitalize on this, partners thought that linking their organization’s websites to PreVAiL (and vice versa) would be very useful. Many participants said they valued the ability to call on PreVAiL researchers for information. In this way, participants “*felt liberated to ask for advice*” [P-05], indicating that being part of PreVAiL had provided a new and expedient mechanism to connect with researchers.

“*…we needed to have* [information]*, it’s quite a quick turnaround, so first of all we needed evidence, … so we wrote to a few people* [from PreVAiL] *who provided us with their input and expertise …* [we asked them] *do you have any suggestions from the literature where we could look. And some people had the information on the top of their head, they could send us in different directions and people were very useful.*” [P-05]

Some partners felt their role in PreVAiL was to be an ‘information conduit’ in their own organization. This role of information conduit went both ways: partners bringing ‘PreVAiL knowledge’ to their organizations and partners giving ‘organization knowledge’ to PreVAiL; the latter was often described as providing context regarding the practical realities of organizations.

As PreVAiL was a relatively new network at the time of data collection, many of the PIQ questions relating to the process of knowledge generation (for example, indicators of collaborative research), were found to be inapplicable by partners and researchers. For the question about ‘joint discussion about findings and implications’ on the PIQ, the majority of partners (56%) felt there was joint discussion whereas the majority of researchers (50%) felt the question was not applicable. For those researchers who did respond, many agreed (35%) that there was joint discussion about findings and their implications.

#### Instrumental use of research and knowledge

When participants were asked about how they had used knowledge arising from their partnership with PreVAiL, a variety of responses were given. A few participants talked about using the research instrumentally, for example in presentations. Others reported actively sharing PreVAiL work, such as Research Briefs, with colleagues. One participant explained how a face-to-face meeting (the content, the discussion, and the people) truly shaped the direction of the organization:

“*So the workshop that was added to the Toronto meeting, at first I think they provided direction for the* [organization]*, where we should go with our … surveillance, … it’s actually still not quite done I guess, but it was an important part for us to know what other experts who are moving forward. And since we got quite a broad, you’ve got many different perspectives, that was really useful and the international as well as national and maybe then a very small community within Canada, and everyone basically knows each other, it was useful to get the international perspective.*” [P-05]

Several partners felt that research-focused outputs can be difficult to translate to their own organizations’ immediate needs, but that efforts like the PreVAiL network are a necessary step in a complex process of knowledge-to-action:

“*…when we talk about knowledge translation, a lot of times it’s researchers, it doesn’t necessarily go quite far enough … it might be translation to the next phase of research or scale up or something like that, but … there are people out in communities doing violence prevention every single day and …, we worry some about the fact that they’re not necessarily always using evidence based strategies … what would be the best course of action given this state of the science right now … what would we do right now, if it was our responsibility to you know, implement programs in communities to try to prevent violence, what would we do based on what we know, and I don’t know, I just feel like we have a group, the network that you pulled together is such a, it’s really impressive and is there a way to, to contribute to that dialogue somehow.*” [P-18]

#### Conceptual use of research and knowledge

The majority of participants used PreVAiL-generated knowledge more conceptually – that is, to change or augment their own understanding of violence, resilience, and even data collection and analysis:and

“*I mean there are things that I keep in mind, I think the whole discussion around resiliency for me was very interesting, I can’t say that I have actually done anything in terms of putting into practice but you know, its information that’s circulating through my brain as I’m thinking about other stuff that I’m working on.*” [P-13]

“[T]*he unit never really looked at* [our data] *that way before, I think they looked at it sort of a storage mechanism for process use and didn’t think about the possibilities that they had for analysis, so I think that that kind of way of viewing child protection and looking at it as you know, as a possible source for you know practice information really came out of the project that we started with at the first meeting.*” [P-20]

There were a number of dimensions on the PIQ (Dissemination of Research, Research Findings) that asked about specific ways that participants used knowledge in practice and policy; however, the majority of participants rated these questions as not applicable. This could be attributed to the fact that PreVAiL was still in its early stages, and many of the research findings had not yet been published or made available to team members.

## Discussion

The PreVAiL network, in addition to using an integrated KT, partnership-based model, made an explicit and sustained effort to implement specific KT strategies into its research process through easy-to-use research products, such as research briefs, progress updates, web updates, and regular newsletters. General KT dissemination principles of crafting and targeting key messages, and careful design of how the messages are conveyed were accomplished [38]. Further, efforts were made to enable an authentic IKT process in which knowledge users and researchers were considered true partners [5,39]. Knowledge users were encouraged to collaborate on priority-setting [37], the creation of research questions, and their contributions were sought for other aspects of the research process [39]. Additionally, relationships were explicitly built and sustained through interactions such as face-to-face meetings, telephone conversations, and emails [40].

A common proposition for integrated KT strategies is that the more involved partners are throughout the research process, the better the partnership will be [3,12,15]. Mitchell et al. [12] argue that the idealization of maximum involvement of knowledge users in the research process is narrow and fails to acknowledge a diversity of approaches that may be effective for integrating research into practice. It also seemed that while partners and researchers did not always have common goals, this was not necessarily problematic because members used the network for different purposes and variations in goals would be expected. For example, while many partners wanted to become more involved with PreVAiL, others were already concerned by information overload and were interested in more limited communication tailored to their narrower, more specific aims. This finding perhaps contradicts a widely-held assumption that knowledge users are a homogenous group with similar research-oriented needs, despite the fact that they come from different types (government, NGO, etc.) and levels (e.g., regional, international) of organizations with different capacities to recognize and respond to varying information needs [3], even when they share a common content area. It might also speak to different conceptualizations of what it means to ‘collaborate’ as a partner in research processes; some partners situate themselves as true ‘knowledge users’, i.e., recipients of research findings or recommendations at the end of the research process. Others were and are more actively ‘integrated’ in the process, and are willing to help set priorities, interpret data, and generally engage more fully. Our approach is to allow partners to decide what works best for their organization and opt into (or out of) specific activities that meet their needs at any given time. This has, for the most part, led to greater buy-in and longitudinal engagement among partners. This was especially important given the breadth of partner representation – for example, 7 of the original 19 partner organizations were non-Canadian, providing a critical mass that emerged as a strength of the network, ensuring that national priorities were considered in global context. While there were occasions where specific liaisons from partner organizations were not able to participate in meetings or other activities, this did not follow a clear pattern regarding type of organization (NGO, government, national, international). Similarly, some partners have become less involved as their own priorities change, or due to turnover in the liaison position. Allender et al. [41] state that large, international networks like PreVAiL, present different levels of participation from members, and that member roles are not static but shift over time; we agree, and argue that more thought needs to be given to alternate paradigms of IKT that encompass different partner roles, expectations, and levels of involvement.

IKT strategies are thought to lead to the strong application of research findings because the two distinct research-practice communities have been bridged. There was some initial evidence of conceptual and instrumental application of knowledge by partners. However, it was apparent that KT, particularly in relation to knowledge user partners within PreVAiL, did not go ‘far enough’ or ‘maximize its potential’. It was felt that research outputs that have more practical outcomes, recommendations, and specific tools or resources are what can be most useful. This was generally contextualized with the realization that, as a new network, PreVAiL had yet to ‘produce’ much new knowledge, but it certainly flags this as an issue for ongoing work in creating and sharing usable knowledge products. In addition, these processes are set in the context of our specific research area – family violence – which poses particular challenges for KT due to the subject matter, its complexity, specific areas of debate and contestation, and the myriad of potential knowledge users involved [19,42].

However, it was felt by some partners that the process was still too research-centered and did not provide evidence to influence policy in real time. It was also not clear that efforts to increase communication mechanisms or further involve knowledge users in the research process would solve this problem. It has been suggested that, to apply research to immediate problems and maximize KT’s potential, we need to start considering the prospect of researcher involvement in the decision-making process [12]. Locating research directly within policy and practice would set research to a decision-making timeline and, rather than focus on the creation of new evidence, this structure would facilitate the use of current evidence to inform solutions to concrete problems. Science Policy Fellowships (see http://ospa.od.nih.gov/fellowship.html or http://www.cihr-irsc.gc.ca/e/43553.html), where researchers are situated within policy environments, are a recent attempt in this direction. Our study findings suggest a larger but related issue that has been under-discussed by proponents of engaged scholarship, i.e.: At what stage can research findings be conveyed to practitioners and policymakers for actionable insights? The rigors of generalizability for science are well-established, and now the rigors of generalizability for practice and policy require further attention. For example, researchers need to reach a certain comfort level with releasing preliminary, potentially actionable findings, for the sake of the greater good, which might mean upsetting the usual process of peer-reviewed, academic publication. However, to mitigate risk to themselves, researchers would have to be assured by those to whom they report (institutions, funders, etc.) that their careers were not damaged by co-prioritizing evidence-for-practice with evidence-to-publish. Of course, ‘early’ sharing of findings would also have to be guided by ethical standards, where the findings themselves were considered robust enough to be trustworthy, and unlikely to change. Guidance on these decisions could be taken from processes set-up for clinical trials regarding early stopping rules for excessive harm or benefit (such as the DAMOCLES Study group [43]). This type of thinking, though, needs to be balanced against the fact that the creation of more and more evidence is not necessarily helpful, but may in fact induce a ‘policy cacophony’ in which there are too many potential options from which to choose [44].

We anticipate additional research findings that can influence policy and service delivery will be generated as the network matures. Here, we report on the quality of partnerships and knowledge utilization at a formative stage of the network lifecycle but are not able to yet fully evaluate the effectiveness of the network in meeting its three main aims. As research in this broader field emerges, it will then be possible and appropriate to compare transnational IKT networks at the same stage of development – two years into development – for insights about supporting optimal performance [45].

Researchers associated with a research/practitioner/policy network for community-based obesity prevention suggest that centralized support, both fiscally and politically at multiple governmental levels, were key factors in establishing their collaboration [41]. It might be said that PreVAiL has political support, given the various policymakers involved with the project, but other structural considerations that influenced the KT approach are worth noting. First, the approach to the core content, i.e., the explicit breaking down of silos between the previously distinct areas of child maltreatment and intimate partner violence, and between a deficit-focused approach and a resilience orientation, was a common value among this group – people wanted to engage across boundaries and jurisdictions (from developed to developing countries) and address the issues of ‘violence across the lifespan’, taking a public health approach. Second, the network was explicitly designed such that a third content area, with its own co-principle investigator, was identified as ‘knowledge translation and exchange’. Thus, we suggest that these careful approaches to finding common ground and dedicating resources to KT are important stabilizers in this network. Future evaluations will determine the extent to which this prediction holds merit.

The findings need to be considered in light of potential study limitations. There was some confusion with some of the PIQ wording arising from the fact that it was originally developed for a two-party partnership (researcher – policymaker) rather than a network partnership. Further, although PreVAiL principal investigators were not involved with data collection, it is possible that social desirability bias inflated some of the interview responses. The first and second authors, PreVAiL members who do not have a history of working in the area of family violence prevention, were responsible for the study design, data collection, and analysis, while the third author – a PreVAiL co-principal investigator – contextualized interpretations of the findings. This approach allowed us to minimize potential bias while working in an integrated KT way. Nevertheless, this study points to several areas of network strengths as well as areas that could use further attention for improved functioning. Almost all participants said that, on an individual level, PreVAiL has provided personal learning in the form of hearing about new research, new knowledge about the evolution in research methods in violence research, and improved connections with Canadian and international experts in violence research. The overall positivity toward PreVAiL on both the PIQ and in interviews, further demonstrates the value that PreVAiL is providing to individuals, various fields of research, and the broader community.

## Conclusions

We believe the findings are applicable to other transnational network-oriented partnerships where a diversity of stakeholders – both from multiple research disciplines, as well as from multiple policy, advocacy, and practice sectors – are brought together around issues of common interest and importance. The findings presented here can therefore be instructive for emerging networks that share some or all of these characteristics – an evolving model for solving complex and intransigent problems. Future evaluations will assess the quality of the network partnerships in association with research outputs, policies, and changes in practice among service organizations.

## Abbreviations

IKT: Integrated knowledge translation; KT: Knowledge translation; PIQ: Partnership indicators questionnaire; PreVAiL: Prevention of violence across the lifespan.

## Competing interests

The authors declare that they have no competing interests.

## Authors’ contributions

AK and NW conceptualized the study, reviewed the analyzed data, and interpreted study findings. SS collected and analyzed the data in consultation with NW. AK wrote the first draft of the manuscript, and all authors provided important feedback to subsequent iterations. All authors approved the submitted version of the manuscript.

## Supplementary Material

Additional file 1 **PreVAiL Partnership Indicators Questionnaire Frequencies.** This frequency table displays full results of the PreVAiL Partnership Indicators Questionnaire, for all team members, and broken down by member category (researcher and partner). Click here for file
