# Supplementary material for: Evaluation of partnerships in a transnational family violence prevention network using an integrated knowledge translation and exchange model: a mixed methods study
Source: Health Res Policy Syst. 2014 May 23;12:25. doi: 10.1186/1478-4505-12-25 (PMC4035665; doi:10.1186/1478-4505-12-25)
Supplement: Additional file 1 — PreVAiL Partnership Indicators Questionnaire Frequencies. This frequency table displays full results of the PreVAiL Partnership Indicators Questionnaire, for all team members, and broken down by member category (researcher and partner). [file 1478-4505-12-25-S1.docx]

# Additional File 1: PreVAiL Partnership Indicators Questionnaire Frequencies

| **Dimensions of**  **Partnerships** | **Indicators** | **1 = Strongly disagree** | **2 = Disagree** | **3 = Neither agree nor disagree** | **4 = Agree** | **5 = Strongly agree** | **6 = Not applicable** | **No Answer** |
| --- | --- | --- | --- | --- | --- | --- | --- | --- |
| **Communication** | - 1. **Communication is on-going. (All)** | 3(8.3) | 2(5.6) | 2(5.6) | 9(25) | 14(38.9) | 5(13.9) | 1(2.8) |
|  | 1.1 Communication is on-going (P) | 1 (11.1) | 1 (11.1) | 0 | 3(33.3) | 4 (44.4) | 0 | 0 |
|  | 1.1 Communication is on-going (R) | 2(7.7) | 1(3.8) | 2(7.7) | 6(23.1) | 10(38.5) | 4(15.4) | 1(3.8) |
|  | - 1. **Communication involves face-to-face meetings as well as telephone, mail, email, and fax methods. (All)** | 2(5.6) | 3(8.3) | 1(2.8) | 14(38.9) | 11(30.6) | 5(13.9) | 0 |
|  | 1.2. Communication involves face- to –face meetings as well as telephone, mail, email and fax methods. (P) | 0 | 1(11.1) | 0 | 3(33.3) | 4(44.4) | 1(11.1) | 0 |
|  | 1.2. Communication involves face-to-face meetings as well as telephone, mail, email and fax methods. (R) | 2(7.7) | 2(7.7) | 1(3.8) | 11(42.3) | 7(26.9) | 3(11.5) | 0 |
|  | - 1. **The same contact people continue over the life of the project. (All)** | 0 | 0 | 4(11.1) | 10(27.8) | 13(36.1) | 8(22.2) | 1(2.8) |
|  | 1.3. The same contact people continue over the life of the project. (P) | 0 | 0 | 0 | 3(33.3) | 6(66.7) | 0 | 0 |
|  | 1.3 The same contract people continue over the life of the project. (R) | 0 | 0 | 4(15.4) | 7(26.9) | 7(26.9) | 25(96.2) | 1(3.8) |
|  | - 1. **A common language/lexicon is used by all parties. (All)** | 1(2.8) | 8(22.2) | 8(22.2) | 7(19.4) | 7(19.4) | 5(13.9) | 0 |
|  | 1.4. A common language/lexicon is used by all parties. (P) | 1(11.1) | 2(22.2) | 3(33.3) | 1(11.1) | 1(11.1) | 1(11.1) | 0 |
|  | - 1. A common language/Lexicon is used by all parties. (R) | 0 | 5(19.2) | 5(19.2) | 6(23.1) | 6(23.1) | 4(15.4) | 0 |
|  | - 1. **Roles, expectations, and criteria for deliverables are explicit.(All)** | 0 | 5(13.9) | 6(16.7) | 7(19.4) | 9(25.0) | 9(25.0) | 0 |
|  | 2.1. Roles, expectations, and criteria for deliverables are explicit.(P) | 0 | 3(33.3) | 1(11.1) | 2(22.2) | 2(22.2) | 1(11.1) | 0 |
|  | 2.1. Roles, expectations, and criteria for deliverables are explicit.(R) | 0 | 2(7.7) | 5(19.2) | 5(19.2) | 7(26.9) | 7(26.9) | 0 |
|  | **3.1 Communication is frequent. (All)** | 5(13.9) | 3(8.3) | 7(19.4) | 11(30.6) | 6(16.7) | 4(11.1) | 0 |
|  | 3.1. Communication is frequent. (P) | 1(11.1) | 1(11.1) | 3(33.3) | 4(44.4) | 0 | 0 | 0 |
|  | 3.1. Communication is frequent. (R ) | 4(15.4) | 2(7.7) | 4(15.4) | 7(26.9) | 6(23.1) | 3(11.5) | 0 |
|  | **4.1 Partners value each other’s contributions. (All)** | 1(2.8) | 0 | 3(8.3) | 8(22.2) | 19(52.8) | 4(11.1) | 1(2.8) |
|  | 4.1. Partners value each other’s contributions. (P) | 0 | 0 | 1(11.1) | 2(22.2) | 5 (55.5) | 1(11.1) | 0 |
|  | 4.1. Partners value each other’s contributions. (R) | 1(3.8) | 0 | 2(7.7) | 5(19.2) | 14(53.8) | 3(11.5) | 1(3.8) |
|  | - 1. **Partners are acknowledged in project documents. (All)** | 0 | 1(2.8) | 2(5.6) | 8(22.2) | 12(33.3) | 12(33.3) | 1(2.8) |
|  | 4.2 Partners are acknowledged in project documents. (P) | 0 | 0 | 1(11.1) | 2(22.2) | 5(55.5) | 1(11.1) | 0 |
|  | 4.2 Partners are acknowledged in project documents. (R) | 0 | 1(3.8) | 1(3.8) | 6(23.1) | 7(26.9) | 10(38.5) | 1(3.8) |
| **Collaborative Research** | - 1. **Joint identification of research questions. (A)** | 1(2.8) | 1(2.8) | 4(11.1) | 10(27.8) | 14(38.9) | 6(16.7) | 0 |
|  | 1.1 Joint identification of research questions. (P) | 0 | 1(11.1) | 0 | 4(44.4) | 3(33.3) | 1(11.1) | 0 |
|  | - 1. Joint identification of research questions. (R) | 1(3.8) | 0 | 4(15.4) | 6(23.1) | 11(42.3) | 4(15.4) | 0 |
|  | - 1. **Each partner’s needs and constraints expressed. (A)** | 1(2.8) | 0 | 7(19.4) | 11(30.6) | 10(27.8) | 7(19.4) | 0 |
|  | 1.2 Each partner’s needs and constraints expressed. (P) | 0 | 0 | 4(44.4) | 1(11.1) | 2(22.2) | 2(22.2) | 0 |
|  | 1.2 Each partner’s needs and constraints expressed. (R) | 1(3.8) | 0 | 3(11.5) | 10(38.5) | 8(30.8) | 4(15.4) | 0 |
|  | - 1. **Joint designing of research protocol. (A)** | 1(2.8) | 1(2.8) | 5(13.9) | 11(30.6) | 7(19.4) | 11(30.6) | 0 |
|  | 1.3 Joint designing of research protocol. (P) | 0 | 1(11.1) | 2(22.2) | 2(22.2) | 2(22.2) | 2(22.2) | 0 |
|  | 1.3Joint designing of research protocol. (R) | 1(3.8) | 0 | 3(11.5) | 9(34.6) | 5(19.2) | 8(30.8) | 0 |
|  | - 1. **If relevant, joint data collection. (A)** | 0 | 0 | 4(11.1) | 2(5.6) | 3(8.3) | 27(75.0) | 0 |
|  | 1.4 If relevant, joint data collection. (P) | 0 | 0 | 1(11.1) | 0 | 0 | 8(88.8) | 0 |
|  | 1.4 If relevant, joint data collection. (R) | 0 | 0 | 3(11.5) | 2(7.7) | 3(11.5) | 18(69.2) | 0 |
|  | **1. 5. If relevant, joint data analysis. (A)** | 0 | 0 | 5(13.9) | 5(13.9) | 5(13.9) | 21(58.3) | 0 |
|  | 1.5 If relevant, joint data analysis. (P) | 0 | 0 | 1(11.1) | 1(11.1) | 1(11.1) | 6(66.6) | 0 |
|  | 1.5 If relevant, joint data analysis. (R) | 0 | 0 | 4(15.4) | 4(15.4) | 4(15.4) | 14(53.8) | 0 |
|  | - 1. **Joint ongoing evaluation of relevance of research (e.g. current project, new findings, new partner needs etc.) (A)** | 0 | 2(5.6) | 2(5.6) | 11(30.6) | 5(13.9) | 15(41.7) | 1(2.8) |
|  | 1.6 Joint ongoing evaluation of relevance of research (e.g. current project, new findings, new partner needs etc.) (P) | 0 | 1(11.1) | 1(11.1) | 3(33.3) | 1(11.1) | 3(33.3) | 0 |
|  | - 1. Joint ongoing evaluation of relevance of research (e.g. current project, new findings, new partner needs etc.) (R) | 0 | 1(3.8) | 1(3.8) | 8(30.8) | 4(15.4) | 11(42.3) | 1(3.8) |
|  | - 1. **Joint discussion of findings and implications. (A)** | 0 | 1(2.8) | 3(8.3) | 6(16.7) | 8(22.2) | 17(47.2) | 1(2.8) |
|  | 1.7 Joint discussion of findings and implications. (P) | 0 | 1(11.1) | 0 | 2(22.2) | 3(33.3) | 3(33.3) | 0 |
|  | - 1. Joint discussion of findings and implications. (R) | 0 | 0 | 3(11.5) | 4(15.4) | 5(19.2) | 13(50) | 1(3.8) |
|  | - 1. **Feedback about research report is provided before final draft. (A)** | 0 | 0 | 3(8.3) | 5(13.9) | 7(19.4) | 20(55.6) | 1(2.8) |
|  | 2.1 Feedback about research report is provided before final draft. (P) | 0 | 0 | 1(11.1) | 2(22.2) | 1(11.1) | 5(55.6) | 0 |
|  | 2.1 Feedback about research report is provided before final draft. (R) | 0 | 0 | 2(7.7) | 3(11.5) | 6(23.1) | 14(53.8) | 1(3.8) |
|  | - 1. **Response to feedback is prompt. (A)** | 0 | 1(2.8) | 1(2.8) | 7(19.4) | 8(22.2) | 18(50) | 1(2.8) |
|  | 2.2 Response to feedback is prompt. (P) | 0 | 0 | 0 | 3(33.3) | 3(33.3) | 3(33.3) | 0 |
|  | - 1. Response to feedback is prompt. (R) | 0 | 1(3.8) | 1(3.8) | 4(15.4) | 5(19.2) | 14(53.8) | 1(3.8) |
|  | - 1. **Only a few rounds of revisions before deliverable is acceptable to all. (A)** | 0 | 1(2.8) | 2(5.6) | 4(11.1) | 4(11.1) | 23(63.9) | 2(5.6) |
|  | 2.3 Only a few rounds of revisions before deliverable is acceptable to all. (P) | 0 | 0 | 0 | 1(11.1) | 1(11.1) | 6(66.7) | 1(11.1) |
|  | 2.3 Only a few rounds of revisions before deliverable is acceptable to all. (R) | 0 | 1(3.8) | 2(7.7) | 3(11.5) | 3(11.5) | 16(61.5) | 1(3.8) |
|  | - 1. **Feedback is given after the final deliverable is received. (A)** | 1(2.8) | 0 | 6(16.7) | 1(2.8) | 2(5.6) | 25(69.4) | 1(2.8) |
|  | 2.4 Feedback is given after the final deliverable is received. (P) | 0 | 0 | 2(22.2) | 0 | 0 | 7(77.7) | 0 |
|  | 2.4 Feedback is given after the final deliverable is received. (R) | 1(3.8) | 0 | 4(15.4) | 1(3.8) | 2(7.7) | 17(65.4) | 1(3.8) |
|  | - 1. **Stakeholders and government**   **partners received relevant documents(P)** | 0 | 0 | 1(2.8) | 6(16.7) | 7(19.4) | 21(58.3) | 1(2.8) |
|  | 1.1 Stakeholders and government partners received relevant documents(P) | 0 | 0 | 0 | 1(11.1) | 3(33.3) | 5(55.5) | 0 |
|  | 1.1 Stakeholders and government partners received relevant documents (R) | 0 | 0 | 1(3.8) | 5(19.2) | 4(15.4) | 15(57.7) | 1(3.8) |
|  | **2.1 Recommendations for action reflect current program and policy challenges(A)** | 1(2.8) | 0 | 4(11.1) | 6(16.7) | 3(8.3) | 21(58.3) | 1(2.8) |
|  | 2.1 Recommendations for action reflect current program and policy challenges(P) | 0 | 0 | 1(11.1) | 2(22.2) | 1(11.1) | 5(55.6) | 0 |
|  | 2.1 Recommendations for action reflect current program and policy challenges(R) | 1(3.8) | 0 | 3(11.5) | 4(15.4) | 2(7.7) | 15(57.7) | 1(3.8) |
|  | 3**.1 Presentation formats are similar to those used for other communications within the gov’t (e.g. briefing notes) (A)** | 1(2.8) | 1(2.8) | 5(13.9) | 0 | 2(5.6) | 21(58.3) | 1(2.8) |
|  | 3.1 Presentation formats are similar to those used for other communications within the gov’t (e.g. briefing notes) (P) | 1(11.1) | 1(11.1) | 2(22.2) | 0 | 0 | 5(55.6) | 0 |
|  | 3.1 Presentation formats are similar to those used for other communications within the gov’t (e.g. briefing notes) (R) | 0 | 0 | 3(11.5) | 5(19.2) | 2(7.7) | 15(57.7) | 1(3.8) |
| **Research Findings** | 1.1 **Research findings are presented in policy-related format and language(A)** | 0 | 2(5.6) | 5(13.9) | 4(11.1) | 6(16.7) | 18(50) | 1(2.8) |
|  | 1.1 Research findings are presented in policy-related format and language(P) | 0 | 2(22.2) | 2(22.2) | 0 | 1(11.1) | 4(44.4) | 0 |
|  | 1.1 Research findings are presented in policy-related format and language(R) | 0 | 0 | 3(11.5) | 4(15.4) | 5(19.2) | 13(50) | 1(3.8) |
|  | 1.**2 Implications of findings are understood by all. (A)** | 0 | 0 | 7(19.4) | 5(13.9) | 3(8.3) | 20(55.6) | 1(2.8) |
|  | 1.2 Implications of findings are understood by all. (P) | 0 | 0 | 3(33.3) | 0 | 1(11.1) | 5(55.6) | 0 |
|  | 1.2 Implications of findings are understood by all. (R) | 0 | 0 | 4(15.4) | 5(19.2) | 2(7.7) | 14(53.8) | 1(3.8) |
|  | 1.**3 Documentation of feedback to researchers. (A)** | 0 | 0 | 4(11.1) | 3(8.3) | 7(19.4) | 21(58.3) | 1(2.8) |
|  | 1.3 Documentation of feedback to researchers. (P) | 0 | 0 | 1(11.1) | 1(11.1) | 1(11.1) | 6(66.7) | 0 |
|  | 1.3 Documentation of feedback to researchers. (R) | 0 | 0 | 3(11.5) | 2(7.7) | 6(23.1) | 14(53.8) | 1(3.8) |
|  | - 1. **Senior government staff are aware of research findings. (A)** | 0 | 1(2.8) | 7(19.4) | 3(8.3) | 2(5.6) | 22(61.1) | 1(2.8) |
|  | 1.4 Senior government staff are aware of research findings. (P) | 0 | 1(11.1) | 2(22.2) | 0 | 0 | 6(66.6) | 0 |
|  | 1.4 Senior government staff are aware of research findings. (R) | 0 | 0 | 5(19.2) | 3(11.5) | 2(7.7) | 15(57.7) | 1(3.8) |
|  | **1.5 Research findings are discussed or are reflected in government meeting material and research documents. (A)** | 0 | 1(2.8) | 4(11.1) | 3(8.3) | 2(5.6) | 25 (69.4) | 1(2.8) |
|  | 1.5 Research findings are discussed or are reflected in government meeting material and research documents. (P) | 0 | 1(11.1) | 1(11.1) | 0 | 1(11.1) | 6(66.6) | 0 |
|  | 1.5 Research findings are discussed or are reflected in government meeting material and research documents. (R) | 0 | 0 | 3(11.5) | 3(11.5) | 1(3.8) | 18(69.2) | 1(3.8) |
| **Negotiation** | **1**.**1 Roles and responsibilities are documented.** | 0 | 2(5.6) | 5(13.9) | 9(25) | 8(22.2) | 10(27.8) | 2(5.6) |
|  | 1.1 Roles and responsibilities are documented. (P) | 0 | 0 | 4(44.4) | 1(11.1) | 2(22.2) | 1(11.1) | 1(11.1) |
|  | 1.1 Roles and responsibilities are documented. (R) | 0 | 2(7.7) | 1(3.8) | 8(30.8) | 6(23.1) | 8(30.8) | 1(3.8) |
|  | **1.2 Written terms of reference for research project (or similar document). (A)** | 1(2.8) | 1(2.8) | 2(5.6) | 8(22.2) | 7(19.4) | 16(44.4) | 1(2.8) |
|  | 1.2 Written terms of reference for research project (or similar document). (P) | 1(11.1) | 1(11.1) | 1(11.1) | 1(11.1) | 1(11.1) | 4(44.4) | 0 |
|  | 1.2 Written terms of reference for research project (or similar document). (R) | 0 | 0 | 1(3.8) | 7(26.9) | 6(23.1) | 11(42.3) | 1(3.8) |
|  | **2.1 Requirements for deliverables and time lines are documented. (A)** | 0 | 0 | 6(16.7) | 11(30.6) | 5(13.9) | 13(36.1) | 1(2.8) |
|  | 2.1 Requirements for deliverables and time lines are documented. (P) | 0 | 0 | 4(44.4) | 2(22.2) | 1(11.1) | 2(22.2) | 0 |
|  | 2.1 Requirements for deliverables and time lines are documented. (R) | 0 | 0 | 2(7.7) | 9(34.6) | 4(15.4) | 10(38.5) | 1(3.8) |
|  | **2.2 Partners make their needs explicit (i.e., in terms of accountabilities, priorities, and long term interest). (A)** | 0 | 0 | 5(13.9) | 13(36.1) | 5(13.9) | 12(33.3) | 1(2.8) |
|  | 2.2 Partners make their needs explicit (i.e., in terms of accountabilities, priorities, and long term interest). (P) | 0 | 0 | 2(22.2) | 3(33.3) | 2(22.2) | 2(22.2) | 0 |
|  | 2.2 Partners make their needs explicit (i.e., in terms of accountabilities, priorities, and long term interest). (R) | 0 | 0 | 3(11.5) | 10(38.5) | 3(11.5) | 9(34.8) | 1(3.8) |
|  | **2.3 Partners document the above needs. (A)** | 0 | 1(2.8) | 8(22.2) | 8(22.2) | 4(11.1) | 13(36.1) | 2(5.6) |
|  | 2.3 Partners document the above needs. (P) | 0 | 0 | 4(44.4) | 2(22.2) | 1(11.1) | 2(22.2) | 0 |
|  | 2.3 Partners document the above needs. (R) | 0 | 1(3.8) | 4(15.4) | 6(23.1) | 3(11.5) | 10(38.5) | 2(7.7) |
| **Partnership Enhancement** | **1.1 Key players and senior management, where relevant, are visibly involved and supported(A)** | 1(2.8) | 0 | 5(13.9) | 8(22.2) | 8(22.2) | 13(36.1) | 1(2.8) |
|  | 1.1 Key players and senior management, where relevant, are visibly involved and supported(P) | 0 | 0 | 2(22.2) | 3(33.3) | 3(33.3) | 1(11.1) | 0 |
|  | 1.1 Key players and senior management, where relevant, are visibly involved and supported(R) | 1(3.8) | 3(11.5) | 0 | 5(19.2) | 5(19.2) | 11(42.3) | 1(3.8) |
|  | **2.1 Discussion of potential long-term plans or structure to ensure continuity of relationship. (A)** | 2(5.6) | 0 | 6(16.7) | 7(19.4) | 10(27.8) | 10(27.8) | 1(2.8) |
|  | 2.1 Discussion of potential long-term plans or structure to ensure continuity of relationship. (P) | 0 | 0 | 4(44.4) | 2(22.2) | 2(22.2) | 1(11.1) | 0 |
|  | 2.1 Discussion of potential long-term plans or structure to ensure continuity of relationship. (R) | 2(7.7) | 0 | 2(7.7) | 5(19.2) | 8(30.8) | 8(30.8) | 1(3.8) |
|  | - 1. **Staff with previous linkages with each other are incorporated into partnership. (A)** | 1(2.8) | 0 | 3(8.3) | 10(27.8) | 8(22.2) | 13(36.1) | 1(2.8) |
|  | 3.1 Staff with previous linkages with each other are incorporated into partnership. (P) | 0 | 0 | 1(11.1) | 4(44.4) | 2(22.2) | 2(22.2) | 0 |
|  | 3.1 Staff with previous linkages with each other are incorporated into partnership. (R) | 1(3.8) | 0 | 2(7.7) | 6(23.1) | 6(23.1) | 10(38.5) | 1(3.8) |
|  | **4.1 Discussion of respective organizational realities of research partners. (A)** | 0 | 1(2.8) | 4(11.1) | 8(22.2) | 7(19.4) | 15(41.7) | 1(2.8) |
|  | 4.1 Discussion of respective organizational realities of research partners. (P) | 0 | 1(11.1) | 2(22.2) | 2(22.2) | 2(22.2) | 2(22.2) | 0 |
|  | 4.1 Discussion of respective organizational realities of research partners. (R) | 0 | 0 | 2(7.7) | 6(23.1) | 5(19.2) | 12(46.2) | 1(3.8) |
